# Accurate, Sensitive, and Rapid Detection of Pseudomonas aeruginosa Based on CRISPR/Cas12b with One Fluid-Handling Step

**DOI:** 10.1128/spectrum.03523-22

**Published:** 2023-01-09

**Authors:** Xiaotong Qiu, Xueping Liu, Ruixue Wang, Xiao Ma, Lichao Han, Jiang Yao, Zhenjun Li

**Affiliations:** a State Key Laboratory for Infectious Disease Prevention and Control, National Institute for Communicable Disease Control and Prevention, Chinese Center for Disease Control and Prevention, Beijing, China; b School of Laboratory Medicine and Life Sciences, Wenzhou Medical University, Wenzhou, China; c Shanxi Bethune Hospital, Shanxi Academy of Medical Sciences, Tongji Shanxi Hospital, Third Hospital of Shanxi Medical University, Taiyuan, China; d Department of Clinical Laboratory, Beijing Friendship Hospital, Capital Medical University, Beijing, China; University of Manitoba

**Keywords:** CRISPR, Cas12b, LAMP, *Pseudomonas aeruginosa*, nucleic acid detection, diagnosis, accurate diagnosis

## Abstract

Pseudomonas aeruginosa is a major bacterial pathogen causing nosocomial infections and accounts for morbidity and mortality among patients with cystic fibrosis. An accurate, sensitive, and rapid method to detect P. aeruginosa is critical for the early control of infection and patient management. In this study, we established a P. aeruginosa clustered regularly interspaced short palindromic repeats testing in one pot (CRISPR-top) assay which detected P. aeruginosa with one fluid-handling step in one tube. The reaction was performed isothermally within 1 h; thus, specific instruments were not required. The optimal reaction conditions of this assay were determined to be a temperature of 55°C; working concentrations of 1 μM for the forward inner primer and backward inner primer, 0.5 μM for the loop forward primer and loop backward primer, and 0.25 μM for the forward outer primer and backward outer primer; as well as a 2 μM concentration single-stranded DNA reporter molecules. In terms of specificity, our assay showed 100% inclusivity and exclusivity among 48 strains, including 15 P. aeruginosa clinical isolates and 33 non-P. aeruginosa strains. The limit of detection of our method was 10 copies per reaction mixture. Forty-six human sputum specimens from patients with respiratory symptoms were tested. Using the results of quantitative real-time PCR as the gold standard, our method showed 85.3% (29/34) sensitivity, 100% (12/12) specificity, a positive predictive value of 100% (29/29), and a negative predictive value of 70.6% (12/17). In summary, the P. aeruginosa CRISPR-top assay developed in the present study is a high-efficiency alternative tool for the accurate and rapid detection of P. aeruginosa, especially in resource-limited settings.

**IMPORTANCE** This study reports a P. aeruginosa CRISPR-top assay which can precisely identify P. aeruginosa using nucleic acids from pure cultures or clinical samples in one pot with one fluid-handling step. The P. aeruginosa CRISPR-top reaction is suitable for on-site testing, and its diagnostic performance can be compared with that of qPCR.

## INTRODUCTION

Pseudomonas aeruginosa, a Gram-negative aerobe, is a major bacterial pathogen causing nosocomial infections. In the United States, P. aeruginosa is the leading cause of ventilator-associated pneumonia in long-term care hospitals and the second most common cause in intensive care units (1). In China, P. aeruginosa is the third most common infection in adults with pneumonia (2). In addition, chronic lung colonization by P. aeruginosa is the major cause of morbidity and mortality among patients with cystic fibrosis (CF) (3). P. aeruginosa also causes urinary tract infection (4), sepsis (5), and skin and subcutaneous tissue infections (6). Recently, the emergence of multidrug-resistant P. aeruginosa, especially carbapenem-resistant P. aeruginosa, has become a challenge for global health (7). Therefore, accurate and rapid detection of P. aeruginosa is critical for the early control of infection and patient management.

To date, the routine detection and identification of P. aeruginosa has relied on microbiological culture in most laboratories. However, the conventional culture-based identification methods, for example, biochemical identification and matrix-assisted laser desorption ionization–time of flight mass spectrometry (MALDI-TOF MS), are time-consuming (>2 days) and dependent on professional operation and laboratory apparatus (8). Thus, the traditional detection method is not suitable for rapid diagnosis. Although quantitative real-time PCR (qPCR) technology is not culture dependent, has been proven to be a valuable tool to detect P. aeruginosa, and is superior to culture-based methods (9), it still requires expensive and sophisticated thermal cyclers.

Recently, clustered regularly interspaced short palindromic repeats (CRISPR)/CRISPR-associated (CRISPR/Cas) systems have paved the way for next-generation nucleic acid detection. Several CRISPR-based diagnostic platforms, for example, CRISPR/Cas12a-based DNA endonuclease-targeted CRISPR *trans* reporter (DETECTR) and CRISPR/Cas13a-based specific high-sensitivity enzymatic reporter unLOCKing (SHERLOCK) have been used to detect multiple pathogens and single-nucleotide polymorphisms, showing single-base mismatch specificity and attomolar sensitivity (10, 11). These Cas effectors possess unique collateral cleavage activities that transiently cleave fluorophore quencher-labeled single-stranded DNA (ssDNA) or RNA reporter molecules once activated, releasing a fluorescent signal. The use of guide RNA (gRNA) makes CRISPR/Cas-based detection extremely specific. However, most CRISPR-based detection platforms require two separate fluid-handling steps, namely, preamplification and CRISPR-based detection steps, resulting in multiple operations with the potential for contamination (12–14). More recently, a one-pot, one-step CRISPR-based nucleic acid detection technology, named CRISPR-top, has been successfully devised and applied to the diagnosis of coronavirus disease 2019 (15). AapCas12b, a thermophilic RNA-guided endonuclease from the type V-B CRISPR/Cas system of *Alicyclobacillu acidophilus*, works at temperatures from 37°C to 60°C (16), which overlaps with the thermal profile of the *Bst* 2.0 enzyme in loop-mediated isothermal amplification (LAMP) at 55°C to 60°C (17). The CRISPR-top technology employs AapCas12b and couples LAMP and the Cas12b reaction in one pot to achieve accurate detection of pathogens, thus avoiding the cross-contamination caused by multiple steps.

**FIG 1:**
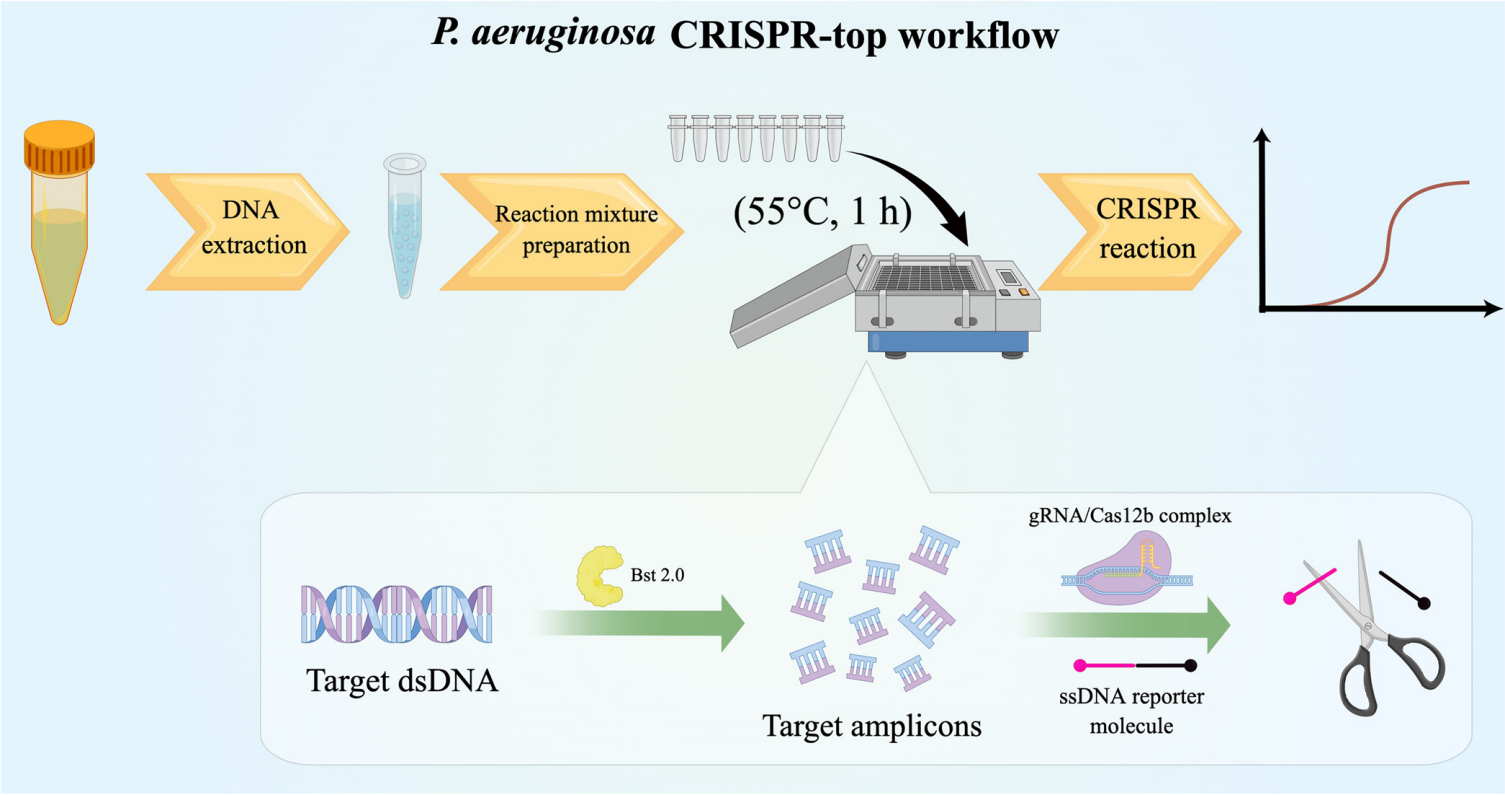
Schematic diagram of P. aeruginosa CRISPR-top workflow (created using Figdraw).

In this study, we applied the CRISPR-top diagnosis platform to establish an accurate, sensitive, and rapid P. aeruginosa detection assay, termed the P. aeruginosa CRISPR-top assay (Fig. 1). *oprL*, a P. aeruginosa-specific and conserved gene encoding a peptidoglycan-associated lipoprotein (18), was selected as the target gene. The optimal reaction conditions of the P. aeruginosa CRISPR-top assay were determined, and its diagnostic performance was evaluated using clinical specimens and compared with that of qPCR.

## RESULTS

### Confirmation of LAMP primers.

To confirm the validity of the primers (Table 1), LAMP reactions were performed at 60°C for 1 h. A total of four reaction mixtures, including one with 10 ng/μL ATCC 27853 genomic DNA, one with 10 pg/μL ATCC 27853 genomic DNA, and two with deionized water (DW), were tested. The turbidities of the reaction mixtures containing 10 ng/μL or 10 pg/μL P. aeruginosa genomic DNA began to increase significantly at 21 min and 38 min, respectively, whereas the turbidities of the two negative controls did not increase within 1 h (see Fig. S1 in the supplemental material). Thus, the validity of the LAMP primer set was confirmed.

**TABLE 1:** Primers, gRNA, and ssDNA reporter molecules used in this study

Primer	Type	Sequence (5′–3′)^*a*^	Length
Pa-F3	Forward outer primer	CGCGTAGTGCTGGAAGG	17 nt
Pa-B3	Backward outer primer	GGTTCTGAGCCCAGGACTG	19 nt
Pa-FIP	Forward inner primer	TAGCGCTGAACGGCCTTG**TTC**GGCACCCGCGAGTACA	37 nt
Pa-BIP	Backward inner primer	GCAGGGTGTTTCGCCGTCGTCGTGGCCGGTAG	32 nt
Pa-LF	Loop forward primer	GCACGACGCTCGCCCAGAG	19 nt
Pa-LB	Loop backward primer	GGTAAAGAGCGTCCGGTCG	19 nt
gRNA	Guide RNA	GUCUAGAGGACAGAAUUUUUCAACGGGUGUGCCAAUGGCCACUUUCCAGGUGGCAAAGCCCGUUGAGCUUCUCAAAUCUGAGAAGUGGCACGGCACCCGCGAGUACAAUAU	111 nt
ssDNA reporter molecule	ssDNA reporter molecule	6-FAM-TTATTATTAT-BHQ1	10 nt

a The inserted PAM site (TTC) is in bold.

### Optimal reaction conditions.

**FIG 2:**
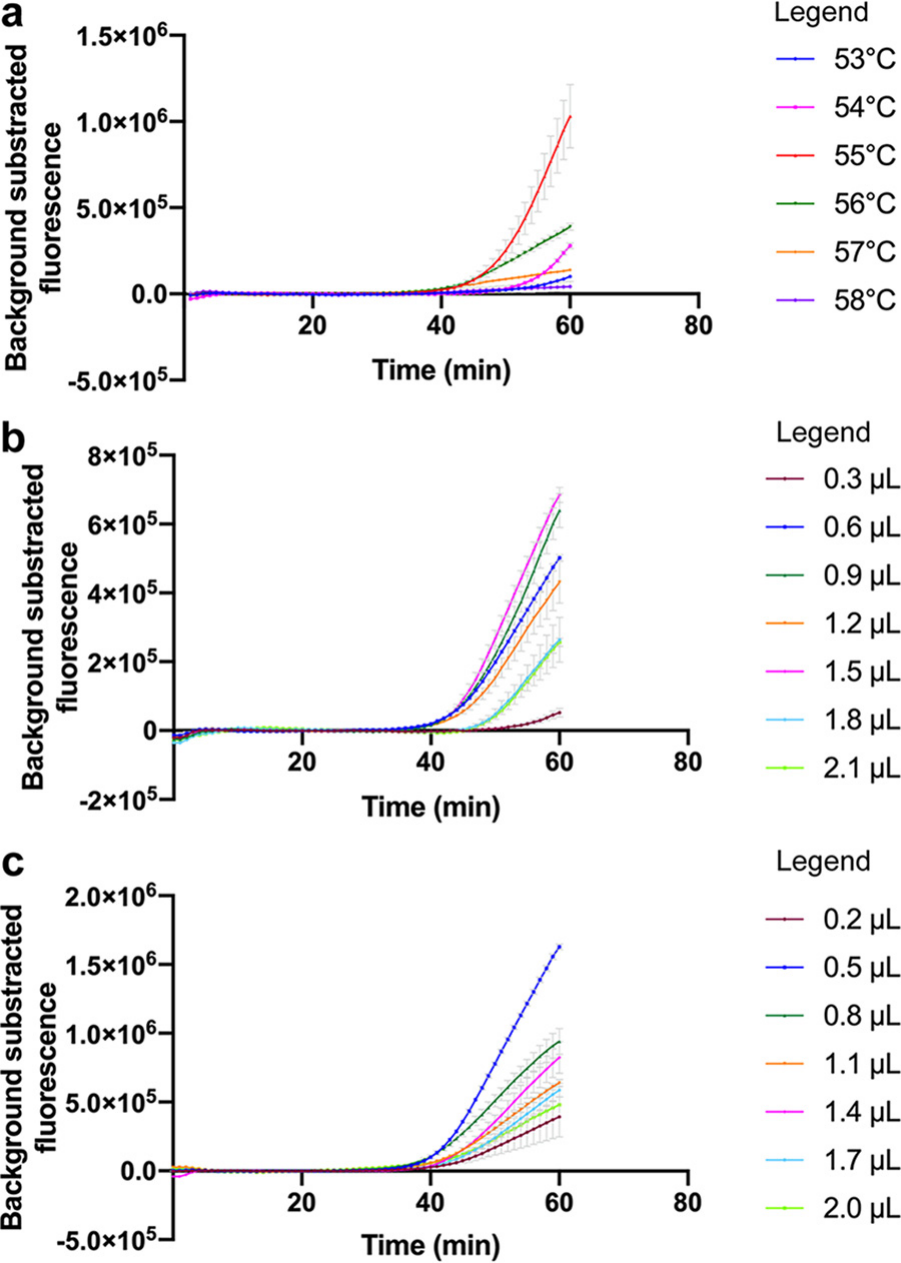
Optimal reaction conditions of the P. aeruginosa CRISPR-top assay. Error bars represent the means ± standard error of means (SEM); *n* = 3 technical replicates. (a) Optimal reaction temperature. The reactions were performed under temperatures ranging from 53°C to 58°C at 1°C intervals. (b) Optimal primer concentration. Serial volumes of LAMP primer premixture (0.3 to 2.1 μL at 0.3-μL intervals) were used to prepare the reaction mixtures. (c) Optimal ssDNA reporter molecule concentration. Serial volumes of 100 μM ssDNA probe (0.2 to 2.0 μL at 0.3-μL intervals) were used to prepare the reaction mixtures.

First, the optimal reaction temperature was determined. Genomic DNA from P. aeruginosa ATCC 27853 was used in each positive reaction and DW comprised the negative control. The reactions were conducted from 53°C to 58°C with 1°C increments. The experiments at each temperature were repeated three times. As shown in Fig. 2a, 55°C was the optimum reaction temperature of the P. aeruginosa CRISPR-top assay, as it produced the highest fluorescence values and the shortest take-off time.

Second, the optimal primer concentration was determined. Six LAMP primers were diluted to 100 μM using DW and premixed as 40 μL each of forward inner primer (FIP) and backward inner primer (BIP), 20 μL each of loop forward (LF) and loop backward (LB) primers, and 10 μL each of forward outer primer (F3) and backward outer primer (B3). Serial volumes of the LAMP primer premixture (0.3 to 2.1 μL, in 0.3-μL intervals) were used to determine the optimal primer concentration for the P. aeruginosa CRISPR-top assay. P. aeruginosa ATCC 27853 and DW served as the positive control and the negative control, respectively. As shown in Fig. 2b, the reaction mixtures containing 0.6 to 1.5 μL of the primer premixture resulted in effective detection. Considering both the cost and effectiveness, 0.9 μL of the primer premixture per reaction was determined as the optimum primer concentration, corresponding to working concentrations of 1 μM FIP and BIP, 0.5 μM LB and LF, and 0.25 μM F3 and B3.

Finally, the optimal ssDNA reporter molecule concentration was determined. The ssDNA reporter molecule was diluted to 100 μM, and serial volumes (0.2 to 2.0 μL, in 0.3-μL intervals) were examined. The positive reactions and the negative control were prepared as described above. As shown in Fig. 2c, 0.5 μL of the ssDNA reporter molecule per reaction mixture, i.e., a working concentration of 2 μM, showed the optimum detection results.

### Specificity and sensitivity of the P. aeruginosa CRISPR-top assay.

**FIG 3:**
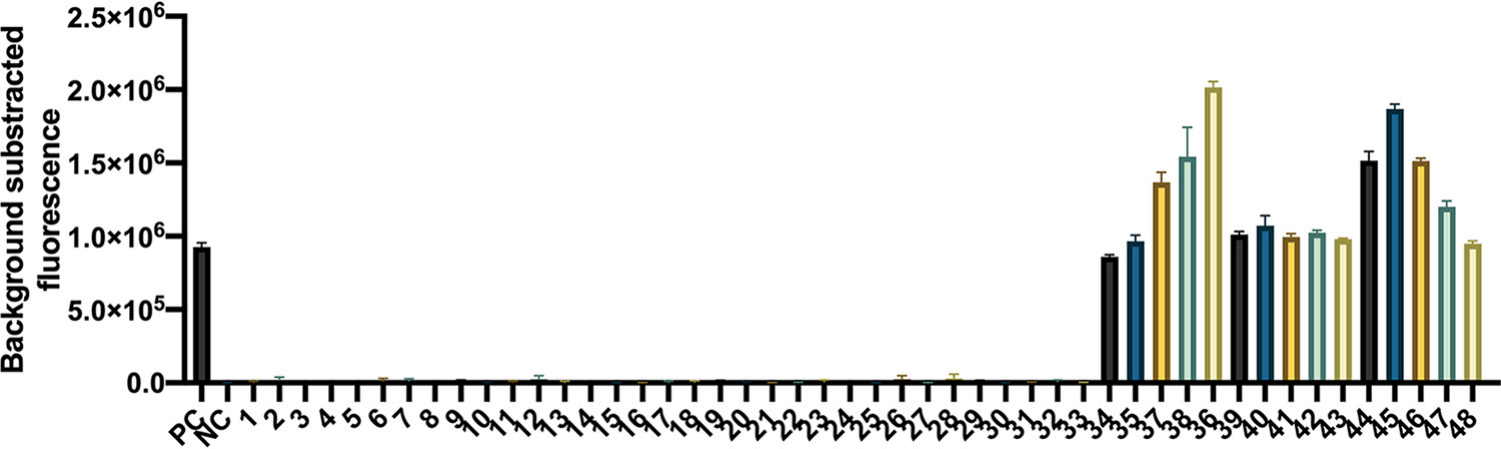
Specificity of the P. aeruginosa CRISPR-top assay. PC, positive control (P. aeruginosa ATCC 27853); NC, negative control (DW). 1, Klebsiella pneumoniae; 2, Acinetobacter baumannii; 3, Streptococcus pneumoniae; 4, Moraxella catarrhalis; 5, Staphylococcus aureus; 6, Escherichia coli; 7, Mycobacterium tuberculosis; 8, Klebsiella aerogenes; 9, Klebsiella oxytoca; 10, Serratia marcescens; 11, Acinetobacter pizzerialum; 12, Acinetobacter junii; 13, Streptococcus mitis; 14, Streptococcus agalactiae; 15, Streptococcus pyogenes; 16, Streptococcus salivarius; 17, Streptococcus oralis; 18, Streptococcus suis; 19, Staphylococcus haemolyticus; 20, Staphylococcus succinus; 21, Staphylococcus epidermidis; 22, Stenotrophomonas maltophilia; 23, Nocardia cyriacigeorgica; 24, Nocardia farcinica; 25, Corynebacterium striatum; 26, Corynebacterium simulans; 27, Corynebacterium propinquum; 28, Corynebacterium aurimucosum; 29, Enterococcus faecalis; 30, Aeromonas caviae; 31, Elizabethkingia anophelis; 32, Ralstonia mannitolilytica; 33, Rothia kristinae; 34 to 48, P. aeruginosa clinical isolates. Error bars represent means ± SEM (*n* = 3 technical replicates).

Forty-eight strains, including 15 P. aeruginosa clinical isolates and 33 non-P. aeruginosa strains (Table S1), were used to verify the specificity of this method. P. aeruginosa ATCC 27853 and DW were used as templates in the positive control and the negative control reactions, respectively. The results revealed that all the P. aeruginosa strains showed positive results, and all the non-P. aeruginosa strains and DW showed negative results (Fig. 3).

**FIG 4:**
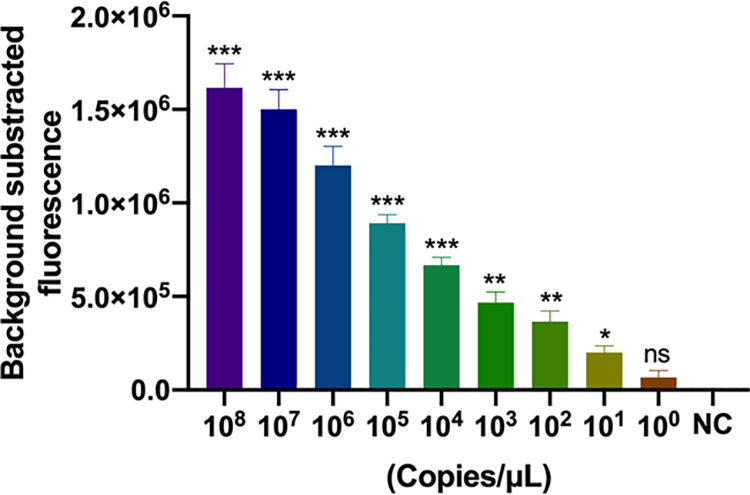
Sensitivity of the P. aeruginosa CRISPR-top assay. Bars represent means ± SEM (*n* = 3 technical replicates). NC, negative control. *, *P < *0.05; **, *P < *0.01; ***, *P < *0.001; ns, no significance (two-tailed Student's *t* test).

In this study, 10-fold serial dilutions of recombinant plasmids containing the targeted fragment of the P. aeruginosa *oprL* gene (from 10^8^ to 1 copies/μL) and DW (negative control) were used to determine the analytical sensitivity of the method. Finally, we determined that this method could detect at least 10 copies per reaction mixture (Fig. 4).

### Application of the P. aeruginosa CRISPR-top assay to clinical samples.

**FIG 5:**
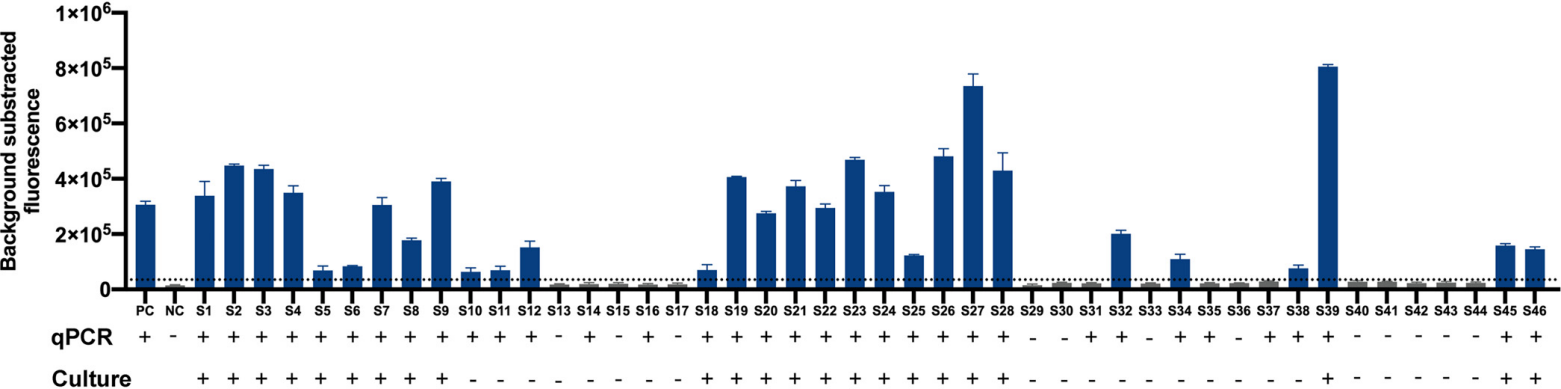
Validation of the P. aeruginosa CRISPR-top assay using clinical samples. The horizontal dashed line indicates the cutoff value set based on the mean + 3 (standard deviations). Bars represent means ± SEM (*n* = 3 technical replicates). PC, positive control; NC, negative control.

A total of 46 human sputum specimens from patients with respiratory symptoms were tested by P. aeruginosa CRISPR-top assay, qPCR, and culture. As a result, 76.1% (35/46) of the samples showed coincident results using all three methods; 13% (6/46) showed positive results by CRISPR-top and qPCR but were negative by culture; and 10.9% (5/46) showed negative results by CRISPR-top and culture but were positive by qPCR. Taking the result of qPCR as the gold standard, the sensitivity and specificity of the P. aeruginosa CRISPR-top assay were 85.3% (29/34) and 100% (12/12), respectively; the positive predictive value and the negative predictive value were 100% (29/29) and 70.6% (12/17), respectively (Table 2). More details of these results are shown in Fig. 5.

**TABLE 2:** Comparison between performance of the P. aeruginosa CRISPR-top assay and qPCR

CRISPR-top result	qPCR		Sensitivity	Specificity	PPV^*a*^	NPV^*a*^
	No. positive	No. negative				
Positive	29	0				
Negative	5	12	85.3%	100%	100%	70.6%
Total (*n* = 46)	34	12				

a PPV, positive predictive value; NPV, negative predictive value.

## DISCUSSION

Several studies have suggested that early detection and adequate treatment are important to prevent or postpone chronic colonization of P. aeruginosa in patients with CF, and molecular detection methods, e.g., qPCR, are superior to culture to detect early colonization (9, 19, 20). However, PCR-based molecular diagnostic techniques require sophisticated instruments, and thus their application is restricted in some resource-limited regions and in the field. CRISPR-based diagnostics were reported as one of seven technologies to watch in 2022 (21). Isothermal amplification coupled with CRISPR-based detection has shown higher sensitivity and specificity than isothermal amplification alone in pathogen detection (22). In this report, a CRISPR-assisted detection method, a P. aeruginosa CRISPR-top assay, was developed. The whole assay process can be completed within 1 h at a constant temperature and without sophisticated instruments, making this method suitable for point-of-care tests.

Optimization of reaction conditions is critical for a detection system to achieve good efficiency. The P. aeruginosa CRISPR-top assay is performed at a constant temperature; thus, a suitable reaction temperature is key to acquiring more effective detection. In this study, the optimal reaction temperature was selected based on the final fluorescence values and the take-off time. Although a positive result can be observed within 40 min, 1 h is still recommended for our assay to achieve maximum sensitivity. Additionally, appropriate working concentrations of the primers and the ssDNA reporter molecule are important. Too much or too little of the oligonucleotides might decrease the effectiveness of detection (Fig. 2b and c), as reported in a previous study (22).

To validate the feasibility of our method in clinical practice, 46 human sputum specimens from patients with respiratory symptoms were tested. qPCR is the gold standard for conventional nucleic acid detection; therefore, we evaluated the consistency of CRISPR-top and qPCR for diagnosis. Our results indicated that the CRISPR-top technique was a potential alternative to qPCR, although the sensitivity of our method was slightly low, which probably resulted from LAMP amplification and CRISPR-Cas12 collateral cleavage occurring simultaneously. Six positive samples tested by our assay were not validated by culture. There may be three explanations. (i) The reaction mixtures were cross-contaminated. This hypothesis, however, can probably be excluded, as all necessary precautions to avoid contamination were taken, and the six “false-positive” samples were also positive when tested using qPCR. (ii) These “false-positive” samples corresponded to a very low inoculum and/or transient colonization (19). The ultrasensitivity of our method means that infection with a low dose of bacteria or transient colonization would be identified by our assay, but not by culture. (iii) The P. aeruginosa strains in the six samples were in a viable but noncultivable (VBNC) state. qPCR has been validated to detect VBNC P. aeruginosa (23); thus, the fact that all six samples were positive by qPCR tends to support this hypothesis. Furthermore, five samples showed positive results by qPCR, whereas they were negative by our CRISPR-based approach and culture. Although we cannot exclude the possibility that the limit of detection (LOD) of qPCR is lower than our method and the five positive qPCR samples corresponded to much lower inoculums, in our previous study (13) qPCR showed a similar LOD to a CRISPR-based assay but generated some false-positive results.

Some isothermal amplification technologies, such as recombinase polymerase amplification (24) and multiple cross-displacement amplification (25), have been applied for rapid and sensitive P. aeruginosa detection; however, previous research indicated that a relatively high rate of false-positive results might occur in the isothermal amplification assay, resulting in misdiagnosis (26, 27). With LAMP, the false-positive results are usually caused by nonspecific primer interactions (26) and aerosol contamination (28). Careful primer design is the key to avoiding false positives. Moreover, combined with CRISPR-based detection, the false-positive results can be alleviated by the complementary base pairing of the gRNA and the target, which is an advantage of CRISPR-based diagnostics (29). Although a simple, low-cost, and ultrasensitive DNA probe based on a lateral flow biosensor (LFB) with CRISPR/Cas12a and LAMP has been developed with proven effectiveness in P. aeruginosa detection, this method has three operation steps, namely, LAMP amplification, Cas12 reaction, and LFB readout (30). After amplification, the LAMP products are transferred to a Cas reaction system, and then the reaction products are tested using LFB. These multiple manual operations might increase the risk of environmental contamination and cross-contamination. In contrast to the existing CRISPR-based diagnosis platform (30), the P. aeruginosa CRISPR-top assay combines LAMP amplification and Cas collateral cleavage in one reaction tube, thereby decreasing the risk of environmental contamination and cross-contamination to the maximum extent, achieving accurate, sensitive, and rapid detection of P. aeruginosa. Additionally, reaction mixture preparation should be strictly separated from sampling and reaction, which is another way to avoid contamination.

Although CRISPR-mediated detection technologies have become powerful molecular diagnosis tools, the preamplification step is controversial. Some scholars have studied amplification-free CRISPR/Cas detection systems to simplify the process and decrease the risk of cross-contamination (31, 32). However, integrating preamplification with detection is another valuable way to solve the issue. Although it has relatively low sensitivity and a long reaction time compared with other two-step CRISPR-Cas detection methods (14, 33, 34), the P. aeruginosa CRISPR-top assay, with only one fluid-handing step, significantly simplifies the manual operations and saves human resources. According to the principle of this method, a fluorescence reader and a lateral flow biosensor can be used to obtain the results (15). Thus, a temperature-controlled small device and portable strips are sufficient for the P. aeruginosa CRISPR-top detection, making it suitable for point-of-care testing. Moreover, P. aeruginosa CRISPR-top detection is low cost. Each reaction costs approximately 3.50 USD; therefore, this assay is suitable for resource-limited settings.

The P. aeruginosa CRISPR-top assay is simple, specific, and of low cost; however, there are a few aspects that could be further refined in the future. More clinical sample types, especially samples from sterile parts, such as blood, pleural effusion, and urine, should be analyzed to provide more clinical diagnostic significance. Additionally, a nucleic acid extraction-free CRISPR-based detection platform could be developed to further simplify operations.

In conclusion, the developed P. aeruginosa CRISPR-top assay is a simple, rapid, and reliable method that can detect P. aeruginosa in one reaction tube and at a constant temperature. This method is comparable with qPCR; thus, it could be a valuable alternative tool for the rapid and accurate detection of P. aeruginosa, especially for on-site testing and in resource-limited settings.

## MATERIALS AND METHODS

### Reagents and instruments.

Wizard Genomic DNA purification kits (catalog number A1125; Promega, Madison, WI, USA) were used for DNA extraction from pure cultures and clinical specimens. DNA isothermal amplification kits (catalog number HT0600; HuiDeXin, Tianjin, China) were used for the LAMP reaction and the P. aeruginosa CRISPR-top assay. AapCas12b enzyme with Cas12b buffer (catalog number HT100008; HuiDeXin) was used for gRNA/Cas12b complex preparation. Premix *Ex Taq* 2× (probe qPCR, catalog number RR390A; TaKaRa, Dalian, China) was used for qPCR detection. A spectrophotometer with fixed wavelengths of 260 and 280 nm (Nanodrop LITE; Thermo Fisher Scientific, Shanghai, China) was used to determine the concentration of double-stranded DNA. A real-time turbidimeter (Loopamp LA-320c; Eiken, Tokyo, Japan) was used to monitor the LAMP products in primer screening. An isothermal metal bath (catalog number 88870004; Thermo Fisher Scientific) was used for gRNA/Cas12b complex preparation. A real-time fluorescence qPCR instrument (QuantStudio 6 Flex; Applied Biosystems, Foster City, CA, USA) was used for qPCR and as the fluorescence reader in the CRISPR-top reaction. MALDI-TOF MS (microflex LRF; Bruker, Bremen, Germany) was used to identify the strains isolated from clinical samples.

### Bacterial strains and DNA extraction.

P. aeruginosa reference strain ATCC 27853 was used to establish the P. aeruginosa CRISPR-top assay. Fifteen P. aeruginosa clinical isolates and 33 non-P. aeruginosa strains were used to verify the specificity of the method. The strains used in this study are listed in Table S1. All strains were stored in 20% (wt/vol) glycerol broth at −70°C and were identified by MALDI-TOF MS before DNA extraction. The concentrations of extracted genomic DNA from these strains were greater than 10 ng/μL, as determined using the Nanodrop LITE spectrophotometer.

### Primer and gRNA design.

**FIG 6:**
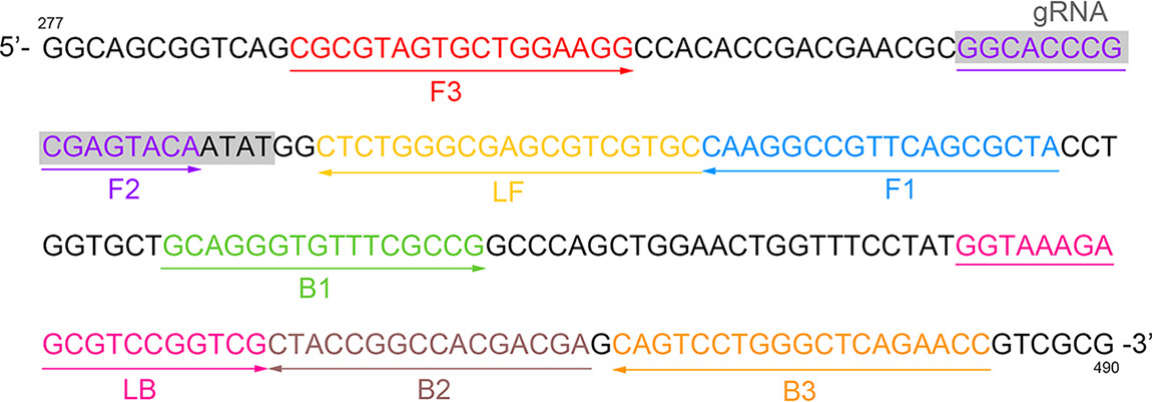
LAMP primers and gRNA design regions. A part of the *oprL* sequence (bp 277 to 490) is shown. Right-pointing arrows and left-pointing arrows indicate sense and complementary strands, respectively.

A LAMP primer set targeting the P. aeruginosa *oprL* gene (GenBank accession number 882991) was designed using Primer Explorer version 5 software (http://primerexplorer.jp/lampv5e/index.html), and a TTC sequence (the standard protospacer adjacent motif [PAM] sequence of AapCas12b) was artificially inserted between the F1c and F2 regions of the forward inner primer (FIP). Oligo Analyzer version 3.1 (Integrated DNA Technologies, Coralville, IA, USA) was used to assess the secondary structure of the primers. The gRNA and ssDNA reporter molecule were designed based on the principle of CRISPR-top (15). The sequences of the LAMP primers, gRNA, and ssDNA reporter molecule are listed in Table 1 and the design sites of the primers and gRNA are shown in Fig. 6. The LAMP primers and the ssDNA reporter molecule (6-fluorescein amidite [6-FAM]/Black Hole Quencher 1 [BHQ1] labeled) were synthesized and purified to high-performance liquid chromatography (HPLC) grade by Sangon Biotech (Shanghai, China). The gRNA was synthesized and purified to RNase-free HPLC grade by GenScript Biotech (Nanjing, China).

### LAMP reaction.

To confirm the feasibility of the LAMP primer set, LAMP amplification was performed at 60°C for 1 h using genomic DNA from P. aeruginosa ATCC 27853 (10 ng/μL and 10 pg/μL) and nuclease-free DW. The 25-μL LAMP reaction mixture contained 12.5 μL of 2× isothermal reaction buffer, 8 units of *Bst* 2.0 DNA polymerase, 1.6 μM (each) FIP and BIP, 0.8 μM (each) LB and LF, 0.4 μM (each) F3 and B3, 1 μL of DNA or DW (negative control), and DW up to 25 μL. The products of LAMP amplification were monitored using the Loopamp LA-320c real-time turbidimeter.

### Standard P. aeruginosa CRISPR-top assay.

First, the gRNA/Cas12b complex was prepared in advance, as described for a previous study (22). In brief, the mixture consisted of 15 pmol AapCas12b and 2 μL of gRNA (10 μM) in Cas12b buffer, which was incubated at 37°C for 10 min. The prepared complex was used immediately or within 12 h (stored at 4°C). Then, the P. aeruginosa CRISPR-top reaction mixture was prepared. The mixture, at a final volume of 25 μL, contained 12.5 μL of 2× isothermal reaction buffer, 8 units of *Bst* 2.0 DNA polymerase, 1 μM (each) FIP and BIP, 0.5 μM (each) LB and LF, 0.25 μM (each) F3 and B3, 2 μM ssDNA reporter molecule, 3.5 μL gRNA/Cas12b complex, 1 μL DNA template or DW (negative control), and DW up to 25 μL. The reaction was performed at 55°C for 1 h and monitored using a fluorescence reader.

### Quantitative real-time PCR.

A qPCR assay was performed using the primers and probe targeting the *oprL* gene, as described previously (19, 35). The qPCR mixture comprised 12.5 μL of 2× Premix *Ex Taq* (probe qPCR), 0.3 μM oprL-F primer (5′-AACAGCGGTGCCGTTGAC-3′), 0.3 μM oprL-R primer (5′-GTCGGAGCTGTCGTACTCGAA-3′), 0.2 μM hydrolysis probe (5′-FAM-TGAGCGACGAAGCC-BHQ1-3′), and 1 μL of DNA template or DW (negative control) and was made up to a final volume of 25 μL with DW. Cycling was performed on the QuantStudio 6 Flex real-time qPCR instrument with an initial hold at 95°C for 10 min, followed by 40 cycles at 95°C for 15 s and 60°C for 1 min (19).

### Specificity and sensitivity of the P. aeruginosa CRISPR-top assay.

Genomic DNAs from 15 P. aeruginosa clinical isolates and 33 non-P. aeruginosa strains (Table S1) were extracted and used to determine the specificity of the assay. Among the 33 non-P. aeruginosa strains, pneumonia-related pathogenic bacteria (for example, Klebsiella pneumoniae, Streptococcus pneumoniae, Staphylococcus aureus, and Moraxella catarrhalis) and other common bacteria (for example, Escherichia coli, Staphylococcus epidermidis, Enterococcus faecalis, etc.) were included. P. aeruginosa ATCC 27853 and DW served as the positive control and the negative control, respectively.

To determine the sensitivity of the P. aeruginosa CRISPR-top assay, the targeted 200-bp fragment of the *oprL* gene was cloned into a pUC57 vector to construct a standard plasmid (33). The copy number of DNA molecules was calculated according to the following equation: DNA copy number (in copies per microliter) = [6.02 × 10^23^ × DNA concentration (in nanograms per microliter) × 10^−9^]/[plasmid length (in base pairs) × 660].

Ten-fold serial dilutions of standard plasmids (from 10^8^ to 1 copies/μL) and DW (negative control) were used as the templates to determine the LOD of this assay. The experiments were repeated three times.

### Evaluation of effectiveness of the P. aeruginosa CRISPR-top assay using clinical samples.

To evaluate the effectiveness of the P. aeruginosa CRISPR-top assay toward clinical samples, 46 human sputum specimens from patients with respiratory symptoms were collected from two hospitals in China. Each sputum specimen was inoculated on a Columbia blood agar plate, a chocolate agar plate, and an eosin-methylene blue agar plate, and plates were incubated at 35°C for 24 to 72 h. The isolated colonies from the sputum samples were identified using MALDI-TOF MS. To prepare DNA templates, 1 mL each of sputum sample was first digested using 4% sodium hydroxide solution, and then the total DNA was extracted using the DNA purification kit according to the technical manual. The sputum samples were stored at −70°C until nucleic acid extraction. The same DNA templates were used for the P. aeruginosa CRISPR-top testing and qPCR.

### Ethics statement.

This study was approved by the Research Ethics Committee of National Institute for Communicable Disease Control and Prevention, Chinese Center for Disease Control and Prevention (permit number ICDC-2019015). All experiments were performed according to relevant regulations.

### Data availability.

The data that support the findings of this study are available from the corresponding author upon reasonable request.
