# Supplementary material for: Accurate, Sensitive, and Rapid Detection of Pseudomonas aeruginosa Based on CRISPR/Cas12b with One Fluid-Handling Step
Source: Microbiol Spectr. 2023 Jan 9;11(1):e03523-22. doi: 10.1128/spectrum.03523-22 (PMC9927138; doi:10.1128/spectrum.03523-22)
Supplement: Supplemental file 1 — Table S1; Fig. S1. Download spectrum.03523-22-s0001.pdf, PDF file, 0.1 MB [file spectrum.03523-22-s0001.pdf]

Supplementary materials for

Accurate, sensitive, and rapid detection of *Pseudomonas aeruginosa* based on CRISPR/Cas12b with one fluid-handling step

Table S1. Strains used in this study.

Bacteria	Strain type (Strain ID)	No. of strains
Pseudomonas aeruginosa	Reference strain (ATCC 27853)	1
	Isolated strain	15
Klebsiella pneumoniae	Reference strain (ATCC 700603)	1
Klebsiella oxytoca	Isolated strain	1
Klebsiella aerogenes	Isolated strain	1
Staphylococcus aureus	Reference strain (ATCC 29213)	1
Staphylococcus haemolyticus	Isolated strain	1
Staphylococcus succinus	Isolated strain	1
Staphylococcus epidermidis	Isolated strain	1
Acinetobacter baumannii	Isolated strain	1
Acinetobacter pizzeri	Isolated strain	1
Acinetobacter junii	Isolated strain	1
Moraxella catarrhalis	Isolated strain	1
Escherichia coli	Reference strain (ATCC 25922)	1
Mycobacterium tuberculosis	Reference strain (H37Rv)	1
Streptococcus pneumoniae	Reference strain (ATCC 49619)	1
Streptococcus oralis	Isolated strain	1
Streptococcus salivarius	Reference strain (K12)	1
Streptococcus agalactiae	Isolated strain	1
Streptococcus pyogenes	Reference strain (CGMC 48848)	1
Streptococcus mitis	Isolated strain	1
Streptococcus suis	Reference strain (GZ1)	1
Stenotrophomonas maltophilia	Isolated strain	1
Nocardia farcinica	Reference strain (IFM 10152)	1
Nocardia cyriacigeorgica	Reference strain (DSM 40350)	1
Corynebacterium striatum	Reference strain (ATCC 43751)	1
Corynebacterium simulans	Isolated strain	1
Corynebacterium propinquum	Isolated strain	1
Corynebacterium aurimucosum	Isolated strain	1
Enterococcus faecalis	Reference strain (ATCC 29212)	1
Aeromonas caviae	Isolated strain	1
Elizabethkingia anophelis	Isolated strain	1
Ralstonia mannitolilytica	Isolated strain	1
Rothia kristinae	Isolated strain	1
Serratia marcescens	Isolated strain	1

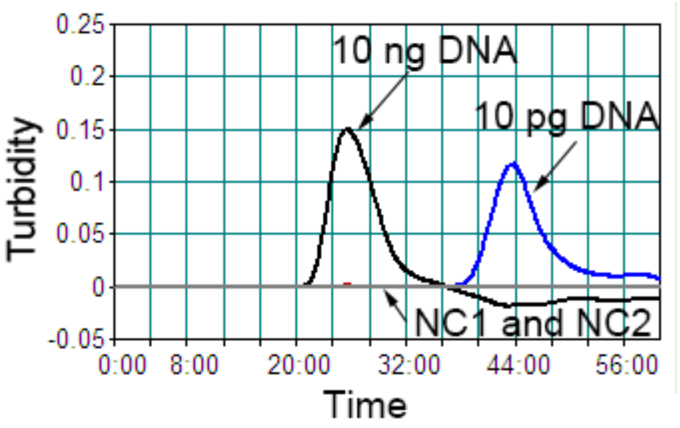

Figure S1. Confirmation of LAMP primers. LAMP reaction was conducted at 60°C for 1 h. *P. aeruginosa* ATCC 27853 genomic DNA and DW were used as the templates of the positive control and the negative control, respectively. NC, negative control.
